# Sherborn’s influence on *Systema Dipterorum*

**DOI:** 10.3897/zookeys.550.9447

**Published:** 2016-01-07

**Authors:** F. Christian Thompson, Thomas Pape

**Affiliations:** 1 Department of Entomology, Smithsonian Institution, Washington, DC 20560, USA; 2 Department of Biosystematics, Natural History Museum of Denmark, Universitetsparken 15, 2100 Copenhagen, Denmark

**Keywords:** Flies, nomenclator, taxonomic catalog, identification, biodiversity informatics infrastructure, quality assurance standard

## Abstract

Flies make up more than 10% of the planetary biota and our well-being depends on how we manage our coexistence with flies. Storing and accessing relevant knowledge about flies is intimately connected with using correct names, and *Systema Dipterorum* provides a single authoritative classification for flies developed by consensus among contributors. The 160,000 species of flies currently known are distributed among 160 recent families and some 12,000 genera, which with their synonyms encompass a total of more than a quarter of a million names. These names and their associated classification are shared with relevant global solutions. Sherborn appears to have done remarkably well indexing Diptera names with an overall error rate estimated to be close to 1%.

## Introduction

Flies are ubiquitous and dominant in most terrestrial ecosystems, by their numbers of species as well as by their immeasurable myriads of individuals. Flies come in a multitude of forms and with an exceedingly vast array of life habits and are often considered the ecologically most varied of the insect orders. Fly larvae in particular are ecologically versatile and have adapted to the harshest of habitats, from pools of crude oil and torrential mountain streams to the bacterial soups of pit-latrines and vertebrate carrion. Flies flourish in the highly disturbed environments created by human activities, often reaching nuisance levels, and flies not surprisingly interfere with man in numerous and varied ways. On the dark side, flies dominate among the blood-sucking pests, with some of the most potent of human diseases being transmitted by dipterous vectors, thereby causing suffering that goes beyond description. However, flies are also beneficial, for example through their processing and recycling of the large quantities of surplus organic material produced by many of our modern societies (Pape 2009). Flies are essential for the successful pollination of many flowering plants, including several crops: without flies we would have no chocolate (Young 1994). Flies are important as both specialist and generalist pollinators, and in cooler or more shaded habitats flies are more important pollinators than bees (National Research Council Committee on the Status of Pollinators 2007, Ssymank et al. 2008). Actually, certain flies appear to be such critical pollinators that some authorities apparently think they are bees (O’Toole and Raw 2004, cover photo)!

The significance of flies reaches deep into our culture. Disease-carrying flies have had tremendous impact on local demography and land-use far beyond any other group of insects, and in the case of West African sickle-cell anemia, flies—even if mediated through a parasite—have reached into our very genome by indirectly favoring a specific genetic mutation (Sabeti et al. 2006, Higgs and Wood 2008). Mimetic flower flies enrich our lives by their stunning similarity to various bees and wasps, and *Drosophila* fruit flies tell us about evolution and genetics from preliminary school to the most advanced research in human genetic disorders. Flies may be seen as part of an ‘extended phenotype’ of our civilization, with the archetypical ‘fly’ being embedded as a key element in many old tales and myths (Kohler 1994, Courtney et al. 2009).

Flies are ancient. The earliest flies began diversifying in the Upper Triassic some 225 million years ago (Grimaldi and Engel 2005), and following three episodes of particularly rapid radiations, flies today constitute about 10% of the known (published) biodiversity, i.e., some 160,000 species (Wiegmann et al. 2011). This multitude of species is distributed among 160 recent families and some 12,000 genera (Pape et al. 2011; Pape and Thompson [online]). But estimates suggest that we may know as little as 10% of the species actually existing on our planet, and if that holds for Diptera, there may be one and a half million species of flies occurring worldwide, 90% of which have not yet been named.

## Fly encyclopedia of past and present

What do people want to know about flies? People may be confronted with a fly that is strange to them, so they may want to know: “What is it?”, “What does it do?” and “Where did it come from?” Or they may have a specific problem, for example with rotting oranges, and when learning that a fly maggot is the problem, they may want to know what fly it is. The resolution in each case starts with the identification of the fly, i.e., the first and crucial step leads to a name. With the right name, people get access to knowledge (Thompson 1996).

Knowledge about life (organisms over time), which was first essentially locked up in *Systema Naturae*, is now dispersed across hundreds of thousands of works, but maybe some day soon it will again be unified—or rather interconnected by means of an *Encyclopedia of Life* (http://www.eol.org). To build such an all-encompassing encyclopedia, we must first assemble the critical pieces of the biodiversity informatics infrastructure. Just as what we need for life in a modern society is transported on a system of airways, highways, seaways, and railways, biodiversity information must also be disseminated via a critical infrastructure. That infrastructure starts with nomenclators and catalogs, and the information is mediated by way of names. Information on identification and classification is disseminated through revisions and monographs. Most of the infrastructure of systematics remains in the traditional printed medium, but the migration to the online, digital medium of the Internet has begun. For flies, the first critical component of the biodiversity informatics infrastructure is the *Systema Dipterorum*
(SD: see http://www.diptera.org; Evenhuis, Pape et al. 2010), a nexus of nomenclator and taxonomic catalog.

**Figure 1.**
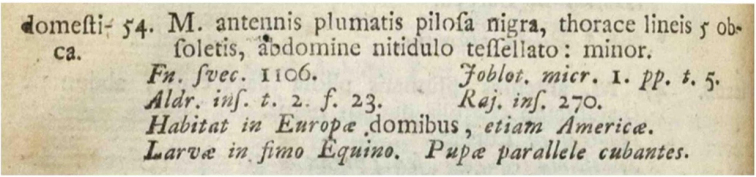
The entry for *Musca domestica* in *Systema Naturae*, 10^th^ edition (Linnaeus 1758).

The past is our prologue as we build on the knowledge of our predecessors and learn from their mistakes. The official start of the modern understanding of flies and their classification has been deemed to be the 10^th^ edition of Linnaeus’ *Systema Naturae* in 1758 (ICZN 1999). Linnaeus produced the first comprehensive database of the natural world. He was first to apply a uniform set of names for all known life forms and to place those names into a “natural” hierarchical classification. Using a fixed suite of formal ranks, he divided the living world into three kingdoms, many classes, and even more orders, genera, and species. Flies were placed in the order Diptera (with some noteworthy exceptions) of the class Insecta. Linnaeus derived both his classification of flies and their name from Aristotle. For the order, the principal character Linnaeus used was the presence of only two wings, but he also noted the halteres, the modified second pair of wings, which is the most conspicuous unique autapomorphy of the dipteran clade. He divided the order into 10 genera and 191 species, and for each species he provided a single word, a specific name or epithet, then a diagnosis, distribution statement, summary of the biology, and references to where further information could be found. For flies, Linnaeus cited the works of 24 different authors. His entry for *Musca domestica* (Fig. 1) provides an example of his method, and this is largely what is still needed today by users of biodiversity information: a single, comprehensive, and authoritative reference work. Because Linnaeus was deemed the first, his nomenclature was by default perfect, but as knowledge of nature increased, the *Systema Naturae* quickly became difficult to maintain. Linnaeus’ last edition of his *Systema* was the 12^th^ (Linnaeus 1767). A German, Johann Friedrich Gmelin, took on the task of summarizing our knowledge of nature and produced a 13^th^ edition of the *Systema* in a number of parts spanning the years 1783 to 1793 (with the flies appearing in Gmelin 1790). However, even before this, the students of Linnaeus had already decided to divide the work among themselves. Of those, Johann Christian Fabricius took responsibility for the insects and produced his *Systema Entomologiae* (Fabricius 1775). His system was identical in form to *Systema Naturae*, but was restricted to just the insects. Yet, as the number of insect species and the knowledge about them increased significantly over time, even this *Systema* on all insects could no longer be maintained. Fabricius realized this and, thus began a series of works devoted to one order each. Toward the end of his life, he provided the *Systema Antliatorum* (Fabricius 1805), devoted just to flies, fleas and a few other sucking insects, and in that work Fabricius changed the name of the order from Diptera to Antliata. Again, the format was the same as in Linnaeus’ *Systema*, but with much more knowledge summarized. In 1805 Fabricius knew 1,151 species of flies, distributed among 78 genera as based on his own work and that of 46 other authors (Table 1). Unfortunately, Fabricius was not comprehensive as he did not include everything that was known at the time. Some 1,767 names were missing, represented in the works of 49 missing authors. After Fabricius, a growing cadre of naturalists took on the study of flies, and the number of species and names increased exponentially.

**Table 1.** Number of valid genera and valid species as well as total number of names for the three major Diptera nomenclators through time (fossils excluded).

	Linnaeus 1758	Fabricius 1805	*Systema Dipterorum* 2013
**Genera**	10	78	12,073
**Species**	191	1,151	160,042
**Names**	201	1,242	272,029
**Authors**	24	46	5,701

Comprehensive works like the *Systemae* of Linnaeus and Fabricius fell victim to the rapid increase in biodiversity information. We do understand what we have lost, and using modern technologies (computers and the Internet), we have begun to build their modern equivalents (e.g., Encyclopedia of Life, Species2000, ZooBank; see also Pyle 2016).

What information sources are currently available to help us build the new online *Systema Dipterorum*? As knowledge expanded and became larger than what one person could assimilate, as happened to Linnaeus and Fabricius, the universe of knowledge was subdivided into smaller, more manageable shares. This division was based on different approaches, some divided up the universe by the taxon, others by time or geography or people.

Division by taxon is simple: once Linnaeus (and then Gmelin) did all organisms, then Fabricius did all arthropods and finally all flies, but after that the division of labor was by geography, with Meigen doing all the flies of Europe and Wiedemann doing all the “exotic” (i.e., non-European) flies. Later, the amount of information became too large for comprehensive works, so a new format (catalogs) were invented and used as a summary of our knowledge, having merely a citation to the basic information. The first catalog for Diptera was produced by Osten Sacken (1858), who summarized knowledge about North American flies. Later Kertész (1902–1910) attempted to do this for the world but never finished. In recent times, dipterists have come together to produce regional catalogs of flies: America north of Mexico (Stone et al. 1965), Americas south of USA (Papavero 1966–1984; Amorim and Papavero 2008), Oriental (Delfinado and Hardy 1973–1977), Afrotropical (Crosskey 1980), Palaearctic (Soós and Papp 1984–1993), and Australasian and Oceanian regions (Evenhuis 1989). Add to that the fossils (Evenhuis 1994). With the continuing increase in papers describing new taxa and refining classifications, some of these regional treatments are greatly out-of-date.

Another division of knowledge was by the publications—works that included knowledge about organisms. One of the first to attempt making a bibliography of all works related to zoology (and geology) was Agassiz (1848–1854). Then Hagen (1862–1863) merely attempted to do all works related to arthropods up to and including the year 1862 (updated and revised by Horn and Schenkling 1928–1929), and he was followed by Derksen and Göllner-Scheiding (1965–1968) (with index by Derksen et al. 1975). These works covered knowledge-sources up to 1900. Evenhuis (1997) provided a selected index to some individual works on Diptera (not articles, but separately published “books”) to 1930.

Just as bibliographies greatly facilitate our access to published works, a main portal to scientific names is embodied by the indexes built for those names. The monographs by Linnaeus and Fabricius were such indexes in addition to being taxonomic tools. After them, however, few comprehensive indexes were developed, and although high-profile initiatives are now underway to dynamically interconnect existing indexes in a way that streamlines the taxonomic enterprise (Pyle 2016), we are still vastly behind in aggregating content in interconnected systems.

## Sherborn’s contribution to dipterology

Sherborn (1902; 1922–1932) was the first to attempt a complete index of all scientific names relating to animal species (Evenhuis 2016). For practical reasons, he broke this task down by time. So, Sherborn first indexed all names published before 1800 and then those before 1850. Earlier zoologists, mainly curators at the British Museum (Natural History), had already agreed to develop and maintain an index of all new names applied to animals—the *Zoological Record*—which since 1864 has been an annual index of new names and taxonomic changes. All scientific names for species include two parts. One for the group that the author(s) feel the species belongs to (the genus-group name), and then the specific name itself (the epithet). Consequently, those genus-group names become a critical core to all scientific names. Hence, zoologists realizing that indexing all animal names would be a colossal undertaking decided to first concentrate on indexing the genus-group names. So, Louis Agassiz, who built the first bibliography of zoological works, also built a nomenclator of all genus-group names in Zoology (Agassiz 1846a,b). He was followed by Marshall (1873), Scudder (1882, 1884), Neave (1920–1996) and Schulze et al. (1920–1954). Sherborn, however, indexed both genus-group names as well as species-group names.

**Figure 2.**
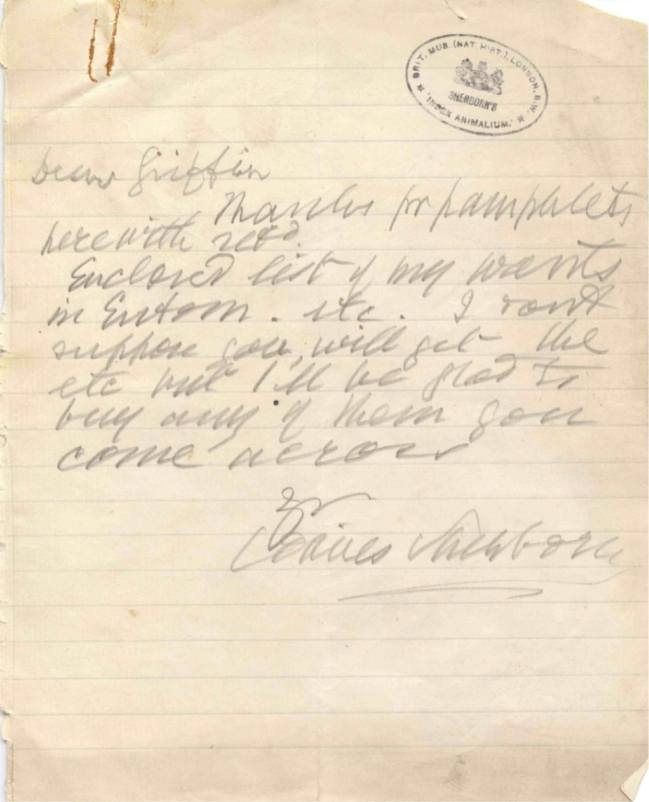
Letter from Sherborn to Griffin accompanying one of his “Want” lists. The text of the message says: “Dear Griffin Thanks for pamphlets herewith recd. Enclosed list of my wants in Entom. etc. I don’t suppose you will get all etc but I’ll be glad to buy any of them you come across. Yours C. Davies Sherborn”.

Today, how do we assess Sherborn’s accomplishments? First, we need to appreciate the platform of nomenclatural legislation that Sherborn worked from. Today we have the 4^th^ edition of the *International Code of Zoological Nomenclature*, but Sherborn worked under the ‘Strickland Code’ (Strickland et al. 1842) and he therefore evaluated names somewhat differently from today’s standards. Second, while today we have computers, database software, etc., Sherborn worked only with slips of paper. Fortunately, Sherborn worked at the British Museum (Natural History) and, therefore, had access to the greatest library of scientific works on animals. Unfortunately, there were works that the British Museum did not have, so Sherborn was partially dependent on colleagues for helping him out for certain names. For example, Francis Griffin helped provide many rare entomological works (Fig. 2). Third, what did Sherborn produce? For each name, Sherborn provided six data elements (name, higher group [genus if species; order if genus], author, source [an abbreviated title and volume], year and page). He checked for two elements that in his view were crucial for validity—publication and diagnosis—and gave four data elements for bibliography (author, year, reference [title, volume, etc.] and place of publication). At the time Sherborn was working, his good friend Bernard Barham Woodward was preparing the catalog of the library of the British Museum (Natural History), so Sherborn (who was helping his friend in the cataloging of the library) decided that certain bibliographic details were unnecessary and unfortunately abbreviated most information.

How good was Sherborn? For a study of the species-group names proposed in the genus *Musca*, Sherborn indexed 1,807 names. Thompson and Pont (1994) found only 3 errors and 1 name that he missed. For all genus-group names proposed for flies (Order Diptera) Sherborn indexed 1,959. We have found another 43 which he missed and another 17 which he missed due to changes in the ICZN (plus a large number of orthographic variants, which by an unfortunate addition to the 4^th^ version of the ICZN are to be deemed unjustified emendations; Evenhuis, O’Hara et al. 2010; O’Hara et al. 2011). Thus, for Diptera Sherborn appears to have done even better than his overall average (Welter-Schultes et al. 2016).

## From *Systema Naturae* to the Web

The impediments that made the continued updating of Linneaus’ original *Systema* impossible were the inflexibility of printing and the increased cost of disseminating knowledge by printing with fixed types and reproducing text in ink on paper. Today, computers are taking over the physical aspects of printing and provide an easy means for integrating the past with current knowledge, and they also allow for alternative dissemination media beyond paper. The Internet with the World Wide Web is a relatively new and ever more dominating medium, allowing anyone anywhere with a computer and online access to receive information in real time from anywhere in the world. The modern workflow in monographic taxonomy is at least potentially greatly enhanced (Penev et al. 2010, Smith et al. 2013), and the challenge is now neither the production nor the media, but what society wants of our science: systematics. [The accepted term for taxonomy and nomenclature and other aspects of our science today is (bio)systematics, a term that is directly derived from *Systema Naturae*. That is, the science of inferring the system inherent in the natural world.]

A Chinese proverb ascribed to Confucius states that wisdom begins with applying the correct names (“*If names be not correct, language is not in accordance with the truth of things*”; cf. Legge 1971). Linnaeus (1737) similarly stated that if you do not know the names, the knowledge about things has no value. Clearly, names and knowledge are intimately connected and work together to create meaningful communication. So, to deliver and decipher biodiversity information about flies, we are first developing the SD as an authority for information about the names of all flies. The names are then organized into a classification (or a taxonomy) just as Linnaeus and Fabricius did in their *Systemae*. For flies, the SD provides a single authoritative classification developed by consensus among contributors and derived from a more comprehensive taxonomy, which includes information on the characters used to generate the scientific hypotheses underlying the classification. The names and their classification are shared with global solutions such as the *Integrated Taxonomic Information System* (http://www.itis.gov/) and *Species 2000 Annual Checklist of Life* (Roskov et al. 2013; http://www.sp2000.org/). A substantial amount of the taxonomic information still remains in the traditional print medium, but the situation is rapidly changing. This is partly because an increasing quantity is now being produced online, with prime examples being serials like *Zootaxa* (http://www.mapress.com/zootaxa), and *ZooKeys* (http://pensoftonline.net/zookeys), but few contemporary journals with taxonomic content lack an online edition, and a growing number are online only, like *Biodiversity Data Journal* (http://www.biodiversitydatajournal.com/) and *European Journal of Taxonomy* (http://www.europeanjournaloftaxonomy.eu/index.php/ejt). Add to that the massive amount of legacy data being digitized through the *Biodiversity Heritage* Library (http://www.biodiversitylibrary.org), *Google® Books* (http://books.google.com), *AnimalBase* (http://www.animalbase.org), and *Gallica* [Bibliotheque National de France] (http://gallica.bnf.fr).

Our survival and well-being ultimately depends on accumulated knowledge about the ‘nuts and bolts’ of the natural world, and perhaps nowhere else in the natural sciences do we find a greater variety of different ‘units’ than in the biological discipline of taxonomy. Ever since Linnaeus, the basic unit of biological classifications is the species, and with our living world containing an estimated 5–12 million species, the need for names obviously is paramount.

**Figure 3.**
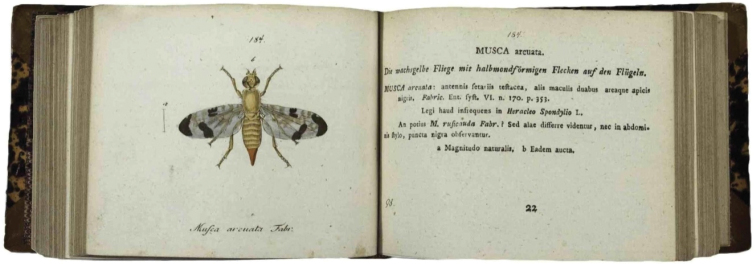
An example of the ‘species pages’ found in Panzer (1792–1813); here his entry for *Musca arcuata* Fabricius, 1781.

Classifications are merely hierarchical groupings, and in evolutionary biology the basic unit is the species. Panzer (1792–1813), at least for entomology, was the first to recognize that if information dissemination is focused on this unit so that each unit is separate and independent from others, then new information can easily be integrated and new classifications easily generated. Panzer did this in the format of small booklets, species-by-species, with text and image on facing pages (Fig. 3). Today this is recognized as the concept of the online species page, some of the first examples of which were placed at the USDA Diptera Website in April 1996 and are now incorporated into the *Encyclopedia of Life* (http://www.eol.org).

The last but most important set of components of the biodiversity infrastructure for users is identification tools. These range from the early paper-based diagnoses provided by Linnaeus and Fabricius to modern interactive, image-rich expert systems that run on hand-held tablets and smartphones, which from being available only for more conspicuous species like birds and whales, now are rapidly expanding to include applets for categories like forest pest insects, tree fungi, mushrooms and broadleaf weeds. For the flies, there are still only a few examples of identification tools that have left paper as the medium for storing and conveying relevant information. Primary examples of CD-ROM based identification systems for flies are the *Fruit Fly Expert Identification System* (Thompson 1999) for the identification of pestiferous fruit fly species, *Agromyzidae of the World* (Dempewolf 2004) for the identification of agromyzids of economic importance, and *On-The-Fly* (Hamilton et al. 2006) for the identification of the families of Australian flies, but keys to local faunas, like the dacine Tephritidae of Malesiana (White and Hancock 2004) and the Asilidae of Germany (Geller-Grimm 2003), are also available. Works accessible through the Internet, and usually by way of the World Wide Web, are of increasing importance because of obvious advantages in easy access and versatility in continuous updating (e.g., Walter and Winterton 2007). Noteworthy examples are the *Canadian Journal of Arthropod Identification* (http://www.biology.ualberta.ca/bsc/ejournal/ejournal.html) with nine of 25 works being devoted to Diptera, the online pages for the pestiferous fruit flies (Carroll et al. 2006a,b), the *Keys to the Medically Important Mosquito Species* (Walter Reed Biosystematics Unit [online]) and *MOSCHweb* (Cerretti et al. 2012) with a matrix-based interactive key to Palaearctic tachinid genera. Additional examples worthy of mention are Dempewolf (2004 [online version], Agromyzidae), Fetzner (2005) and Young (2005) (Tipulidae), and Meiklejohn et al. (2012, Sarcophagidae); see also Winterton (2009).

## *Systema Dipterorum*: today and tomorrow

The SD is designed as a comprehensive online information source for all the critical information about scientific names of flies and the basic information about species of flies. This system grew out of a vision of a group of dipterists who wanted to capitalize on the knowledge that had been generated in preparing a series of regional catalogs of Diptera, which began in the 1960s with a catalog of Nearctic (or rather North American) Diptera that involved Canadian and U.S. fly specialists (Stone et al. 1965). A catalog for the Diptera of the Americas south of the United States was also initiated at this time, but it remained incomplete (Papavero 1966–1984) until revived by Amorim and Papavero (2008) and is now largely completed. The Oriental Diptera were cataloged soon after (Delfinado and Hardy 1973–1977), closely followed by the Afrotropical Diptera (Crosskey 1980). In 1984, at the International Congress of Entomology in Hamburg, dipterists gathered to celebrate the start of the effort to catalog the Palaearctic Region (Soós and Papp 1984–1993), the largest and historically most complex region. Subsequent years focused on completing the cycle with an Australasian/Oceanian catalog (Evenhuis 1989) and starting a series of world catalogs. Funding was successfully obtained from the U.S. National Science Foundation to do that last regional catalog (Evenhuis 1989), and private funding contributed to the production of the world fossil fly catalog (Evenhuis 1994), but attempts to secure funding for further world catalogs have been unsuccessful. USDA provided pilot-test project funds to develop new technologies for an expert identification and information system for fruit flies (Thompson et al. 1993), which provided the basis for the current SD. The original database software used was based on a Wang proprietary COBOL data-management system, and later migrated to FileMaker Pro, the current software system.

The SD is today a fully online system containing all the critical information about the system itself and its contents. What follows here is merely a snapshot of what was available online as of October 2013. Irregularly, the SD (initially as the *BioSystematic Database of World Diptera*) is archived to CD-ROM via the *Diptera Data Dissemination Disk* series (Thompson and Evenhuis 1999, Norrbom and Thompson 2004). As segments of information are completed and peer-reviewed, they have been published in traditional print format in the series *Myia* (e.g., Woodley 2001, Brake and Thompson 2011, Mathis 2011, Mathis and Barraclough 2011, Mathis and McAlpine 2011, Mathis and Sueyoshi 2011). Today, the components online are the nomenclator and reference files and all the appropriate supporting documentation for the system. The only major components not yet online are the species interface and online editing facilities for specialists.

The nomenclator and reference files contain all the essential nomenclatural details as well as minimal species information. For each name, information is provided about the original source and format of the name, correct spelling and type information if the name is available, the nomenclatural and taxonomic status of the name, the distribution, and a link to the original reference. The predecessor of *Systema Dipterorum*, the *BioSystematic Database of World Diptera*
(BDWD), was built incrementally in recognition of the long path to perfection and the need to serve the user community. The BDWD was initially based to a large extent on the published regional Diptera catalogs (and several other major sources as explicitly outlined in our online documentation). While those sources were all of a high quality, they were still secondary (some possibly even tertiary), and as our aim is to ultimately present records checked against their original source by a named authority, most records are still flagged as working records.

The SD continues to be built incrementally so as to provide useful information more quickly than having to wait until it is complete at optimal standards (the sources from which the SD was built are documented online). Each record includes a quality assurance standard indicator (these are also documented online) telling users how complete the record is especially in respect to our ultimate status of taxonomic and nomenclatural peer review by assigned specialists. Records meeting the ultimate level are identified by the name(s) of the specialist(s) and date of review. Currently only about 6% of records meet this highest level, but in reality most records are as good as those already published (that is, the source from which they derived) or better (Table 2).

**Table 2.** *Systema Dipterorum* statistics as of October 2013 indicating number of records and the proportion reaching the quality assurance level at which they are ready for pre-publication peer review.

**Number of records**:
198,258 species-group names (160,042 valid)
23,437 genus-group names (12,073 valid)
32,900 references
**Records compared to original literature**:
29,493 species (~15%)
6,138 genera (~27%)
**Records nomenclaturally and taxonomically reviewed**:
11,509 species-group names (~6%)
2,462 genus-group names (~11%)

With *ZooBank* (Polaszek et al. 2008, Pyle and Michel 2009) growing in capacity and having taken the first steps to become part of the nomenclatural legislation concerning names published in digital works (International Commission on Zoological Nomenclature 2012), a liaison between *ZooBank* and *Systema Dipterorum* within the *Global Names Architecture* (Pyle and Michel 2009) is an obvious next step. Names for the planetary biota are best made available to the user community by a global informatics infrastructure, and Diptera names proposed by practicing taxonomists are already to an increasing extent migrated into *ZooBank* by semi-automated routines of front-end taxonomic journals (Smith et al. 2013). The major task of the team behind *Systema Dipterorum* may increasingly be to provide nomenclatural ‘vetting’ plus taxonomic authority, of which the latter is a quality that can be sustained only by the dipterist community itself.

The planned species interface will differ from the nomenclator only in the way the user can query the information. At present, a user enters a name and the nomenclator returns nomenclatural and taxonomic information about that name. The species interface will allow queries about the species and some of its other attributes, such as distribution and biology. So, one can ask, for example, for a list of all the fruit flies known from Costa Rica or for a list of all the species that are known to attack a certain fruit. The challenge of the species interface will be to determine which attributes users want to query (e.g., Conservation status? Distribution? Economic importance? Hosts? Morphology?) and then encode that information. Today, the nomenclator includes only minimal distributional data for species.

The most important aspect of the whole SD enterprise is our team—the people who have contributed their expertise and labor to build the SD (and before that the BDWD).

The final aspect of the SD is its legal status, which is documented online under ‘How to cite & copyrights’. The critical fact is that SD is a community enterprise built by dipterists for themselves and for all people. So the information is without copyright and is freely available to all. While at various times the master database may have resided physically in some institution, that master was always a product of the SD team and belonged to those people. When the SD first went online, it was hosted by the Smithsonian Institution; later it was transferred to the USDA, and most recently it is served by the Natural History Museum of Denmark. In the future it will keep migrating to the best place that is willing to properly maintain and improve it.
